# Helicity conservation of the Riemann–Silberstein vector

**DOI:** 10.1098/rsos.260497

**Published:** 2026-09-09

**Authors:** Gaetano Diglio, Renzo L. Ricca

**Affiliations:** ^1^ Department of Mathematics & Applications, U. Milano-Bicocca, Milano, Italy; ^2^ Faculty of Science, Beijing U. Technology, 100124 Beijing, People's Republic of China; ^3^ Institute for Sustainability with Knotted Chiral Meta Matter (WPI-SKCM^2^), Hiroshima U., Hiroshima, Japan

**Keywords:** Riemann–Silberstein vector, helicity, Maxwell equations

## Abstract

In this paper, we consider Riemann–Silberstein (RS) helicity (RSH) given by the sum of the electric and magnetic helicities. We show that this quantity is chiral under parity transformation and invariant under duality. In the case of null fields in free space, we prove that the electric field behaves as a material, frozen-in field, and demonstrate the conservation of the electric and magnetic helicities; as a corollary, the conservation of the RSH follows suit. These results can find applications in many areas of current research, from the study of knotted fields in electromagnetism to the applications of Hopfion solutions in chiral meta-matter.

## 1. Introduction

Helicity is a fundamental quantity in describing the topological structure of vector fields, with relevant applications in vortex dynamics, electromagnetism and magnetohydrodynamics. In these contexts, it provides a measure of the geometric and topological complexity of field lines given by their degree of coiling and knotting. Magnetic helicity, defined by

(1.1) $$H_{B}=\int A\cdot B\;dV\;,$$

is a well-known invariant of ideal magnetohydrodynamics [1] that is associated with the conservation of the topology of magnetic field lines [2–4]. A dual formulation of helicity based on the electric field and its vector potential C has been considered in the context of electromagnetism, particularly in the study of knotted field configurations [5], but standard formulations of electromagnetic helicity tend to treat the magnetic and electric helicity contributions separately, and often in formally different contexts. A coherent presentation of their dual role in a unified context, highlighting their common properties and differences, is therefore desirable, and this is the purpose of the present paper. To this end, we can take advantage of the Riemann–Silberstein (RS) formalism that offers a natural representation of the electromagnetic field by combining the electric field E and the magnetic field B into a single complex quantity F=E+iB. This approach suggests the possibility of formulating a unified description of helicity based on a complex representation of the constituent fields by introducing the complex potential G=A+iC, from which the electric and magnetic components emerge as real and imaginary parts. From this perspective, the electric and magnetic helicity can be combined into a more general complex functional, whose real part defines a quantity that will be called RS helicity (RSH).

The aim of this work is to analyse the fundamental properties, gauge dependence and conservation conditions associated with the helicity of the RS vector. Under appropriate assumptions, we show that this quantity admits a conservation law that naturally extends the corresponding properties known for magnetic and electric helicities, considered separately. From a physical perspective, RSH provides a measure of the combined topological structure of the electromagnetic field, describing the geometry and topology of the field lines when the electric and magnetic components are treated as a single complex entity. The analysis is conducted in the context of source-free electromagnetic fields, under appropriate assumptions of regularity and decay at infinity, which guarantee the cancellation of boundary terms in order to highlight the fundamental properties in a clear way.

Although there are natural connections between helicity and the topological structure of fields, given for instance by knotted configurations, this work does not intend to provide a comprehensive treatment of these aspects, nor to directly extend the results to the context of magnetohydrodynamics. Rather, it is intended as a preliminary analysis, which may provide a basis for future developments in these directions. The paper is organized as follows: in the next section, we introduce the RS vector; in §3, we define the RSH and discuss its properties and the properties of an interaction term. In §4, we show that the electric field is a material, frozen-in field, and in §5, we prove the conservation of the RSH through the conservation of its electric and magnetic components. We conclude the discussion with a brief re-elaboration of the results in terms of Faraday's tensor and covariant formulation (§6), and some final remarks in §7.

## 2. The Riemann–Silberstein vector

The idea to consider the electric and magnetic fields as parts of a single vector originates from the work of Riemann on a unified approach to space-time. According to this new approach, Silberstein [6,7] proposed a reformulation of Maxwell's equations combining the electric field E and the magnetic field B (both real functions of space x and time t) into a single bi-vector form F=E+iB, which takes complex values in $C^{3}$. This complexification has the advantage that the standard set of Maxwell's equations in free space (no charges and currents) can be recast in a more compact and elegant form, given by

(2.1) $$\left\{ \begin{array}{clc} & \nabla \cdot F=0\;, & \;\\ & i\partial_{t}F=\nabla \times F\;, & \; \end{array}\right.$$

where for simplicity we set the speed of light c=1 (i being the imaginary unit). A re-formulation of the RS vector in terms of Clebsch variables was then introduced by Bateman [8], highlighting the importance of this presentation in classical and quantum theories of electromagnetism [9,10]. This formulation was successfully applied in general relativity, but it was only with the work of Rañada and collaborators [5,11,12] that a full appreciation of the topological properties of the RS vector was realized.

**Figure 1.**
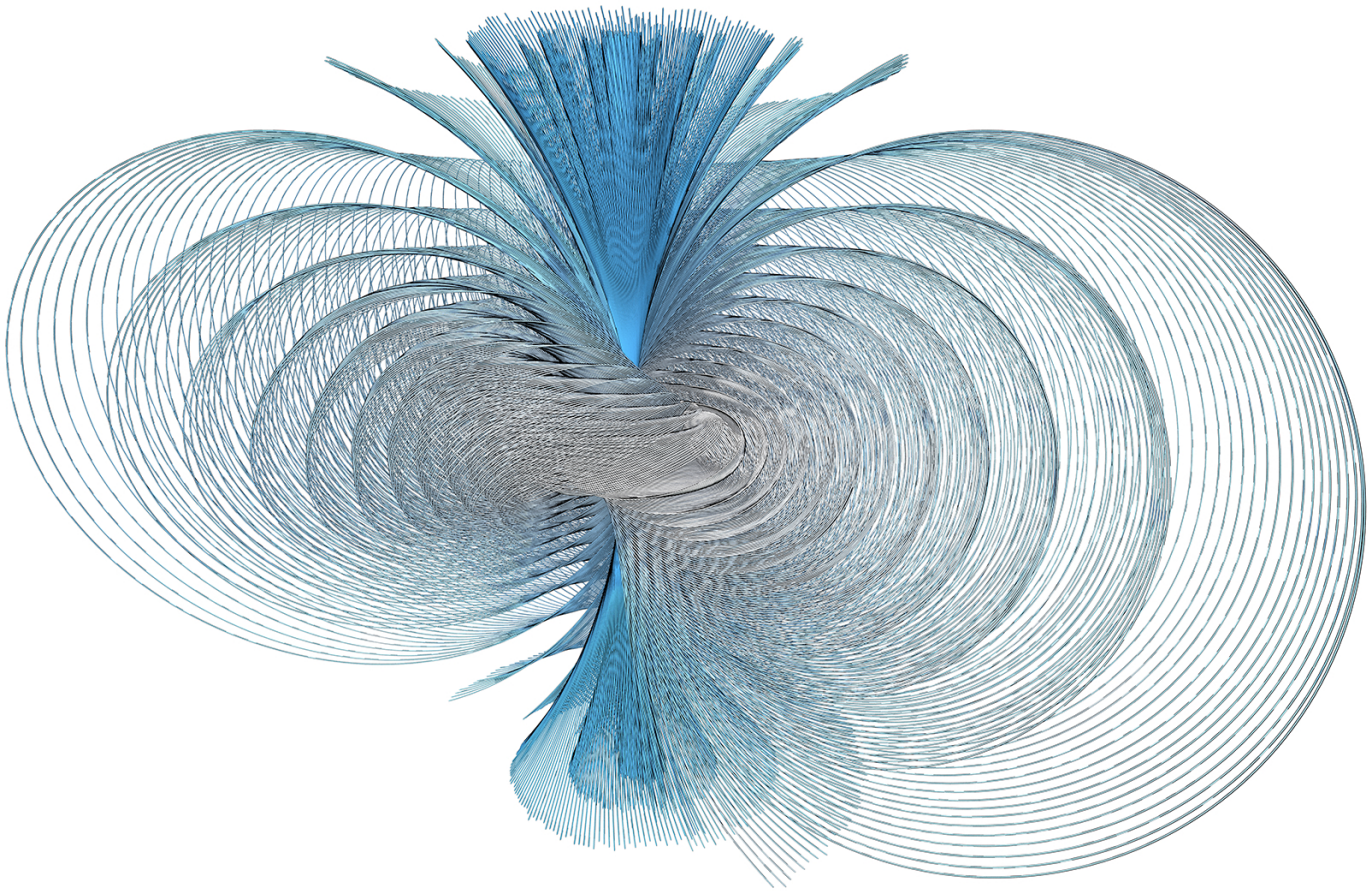
Realisation of a Hopf fibration in $R^{3}$ (courtesy of Oliver Gross).

The electric and magnetic fields are, respectively, given by E=∇×C and B=∇×A, where the potentials C and A are subject to the Coulomb gauge conditions ∇⋅C=0 and ∇⋅A=0. The electric and magnetic helicities are thus defined by

(2.2) $$H_{E}=\int C\cdot E\;dV\;\text{and}\;H_{B}=\int A\cdot B\;dV\;,$$

where integration is over the volume of definition $D\subseteq R^{3}$. For localized fields, the topological interpretation of these quantities in terms of linking numbers has become an important and useful tool for the study of knotted fields [13–16], with applications in many areas of pure and applied science, from the exploitation of Hopfion solutions of radiation fields [17–21] (see figure 1) and spin–orbit interactions in light [22], to the investigations of null fields [23,24] and the analysis of chiral properties in light and soft matter physics [25–27].

## 3. Helicity of the Riemann–Silberstein vector

For our purpose, we consider fields and potentials given by smooth functions f=f(x,t) and localized in a domain D of $R^{3}$. For convenience, we assume D to be a spherical domain of radius R, possibly extended to infinity. All fields are assumed to decay sufficiently rapidly at infinity, and to ensure integral convergence we take $\left\|f\|=O(1/\|x{\|}^{3}\right)$ as x→∞. Let us rewrite the RS vector in terms of vector potentials; we have

(3.1) F=(∇×C)+i(∇×A) .

By introducing the complex-valued potentials, we have

(3.2) $$G=C+iA\;\text{and}\;G^{*}=C-iA\;,$$

where ${}^{*}$ denotes complex conjugate; we can write

(3.3) F=∇×(C+iA)=∇×G .

Let us introduce the quantity

(3.4) $$\begin{array}{rl} H_{F}= & \int G^{*}\cdot F\;dV\;=\int G^{*}\cdot (\nabla \times G)\;dV=\int (C-iA)\cdot [\nabla \times (C+iA)]\;dV\\ = & \int [C\cdot (\nabla \times C)+A\cdot (\nabla \times A)]\;dV+i\int [C\cdot (\nabla \times A)-A\cdot (\nabla \times C)]\;dV\\ = & \;H_{E}+H_{B}+i\int [C\cdot (\nabla \times A)-A\cdot (\nabla \times C)]\;dV\;, \end{array}$$

where $H_{E}$ and $H_{B}$ are the electric and magnetic helicities, respectively, given by equation (2.2). Similarly, we have

(3.5) $$\begin{array}{rl} H_{F^{*}}= & \int G\cdot F^{*}\;dV=\int G\cdot (\nabla \times G^{*})\;dV=\int (C+iA)\cdot [\nabla \times (C-iA)]\;dV\\ = & \int [C\cdot (\nabla \times C)+A\cdot (\nabla \times A)]\;dV+i\int [A\cdot (\nabla \times C)-C\cdot (\nabla \times A)]\;dV\\ = & \;H_{E}+H_{B}+i\int [A\cdot (\nabla \times C)-C\cdot (\nabla \times A)]\;dV\;. \end{array}$$

If we consider the sum

(3.6) $$H_{F}+H_{F^{*}}=2(H_{E}+H_{B})\;,$$

we can introduce the following:

**Definition 1.**

*Given the electric and magnetic helicities defined by equation (2.2), we define the RSH as*

(3.7) $$H_{\text{RS}}=\frac{1}{2}(H_{F}+H_{F^{*}})=H_{E}+H_{B}\;.$$

The real part of $H_{F}$, given by

(3.8) Re(HF)=HE+HB=∫(C⋅E+A⋅B)dV ,

is a measurable physical observable and admits interpretation in terms of the global topological structure of the constituent fields. The term $H_{B}$ (see equation (2.2)) represents the magnetic helicity introduced by Woltjer [1]. In ideal conditions, magnetic helicity is a conserved quantity, and, as shown by Moffatt [2] and Moffatt & Ricca [4], admits topological interpretation in terms of knotting and linking of magnetic field lines. The term $H_{E}$ (see again equation (2.2)), less frequently discussed in literature, arises naturally in dual-symmetric formulations of electromagnetism and contributes, together with its magnetic counterpart, to the definition of a complete electromagnetic helicity (see, for example, the treatment of optical helicity by Bliokh *et al.* [22]).

In this framework, the real part of $H_{F}$ represents a natural generalization of the helicity to an electromagnetic context completely symmetric with respect to its electric and magnetic counterparts. Therefore, as will be shown below, it provides a measure of the overall topological content of the electromagnetic field associated with the intertwined structure of both magnetic and electric fields in free space. Here, $H_{F}$ can thus be interpreted as a natural extension of the classical notion of helicity: its real part captures the standard topological properties of the electromagnetic field, while its imaginary part introduces a coupling term between the electric and magnetic fields, which emerges only in the complex formulation, having no direct analogue in the classical separate treatment of the two contributions.

### 3.1. Interaction term

A new quantity that emerges naturally from the complex formalism adopted via the potential G is the interaction term $I_{int}$, given by the coupling of the electric and magnetic fields:

(3.9) $$\begin{array}{rl} I_{int}= & \;\frac{1}{2i}\{H_{E}+H_{B}+i\int [C\cdot (\nabla \times A)-A\cdot (\nabla \times C)]\;dV\\ & -[H_{E}+H_{B}+i\int [A\cdot (\nabla \times C)-C\cdot (\nabla \times A)]\;dV]\}\\ = & \;\frac{1}{2i}\cdot 2i\int [C\cdot (\nabla \times A)-A\cdot (\nabla \times C)]\;dV\\ = & \int [C\cdot (\nabla \times A)-A\cdot (\nabla \times C)]\;dV=\int (C\cdot B-A\cdot E)\;dV\;. \end{array}$$

This term does not appear in the standard formulation where the two helicity contributions are treated separately, but it emerges naturally in the complex formalism based on the RS field and the complex potential G, reflecting the intrinsically dual nature of the electromagnetic theory in free space. A relevant aspect is the different behaviour with respect to parity transformation. As a consequence of the Coulomb and the Biot–Savart laws (and of the chiral property of the nabla operator), we have

(3.10) x↦−x⇒E↦−E ,C↦C ;B↦B ,A↦−A .

It follows that the contributions A⋅B and C⋅E, and hence the entire real part Re(HF), change sign under parity transformation, according to the chiral character of the magnetic helicity. Conversely, the terms C⋅B and A⋅E remain unchanged, and therefore, the imaginary part Im(HF) given by the last term on the right-hand side of equation (3.9) is under parity transformation even. This highlights the fact that the coupling term is not related to the chirality of the field, but it represents a quantity of different nature, encoding a global interaction between the electric and magnetic components. In this sense, the presence of the imaginary part is a non-trivial consequence of the complex formulation: while the real part naturally extends the classical notion of helicity, the imaginary part introduces a structurally new aspect of the theory, which does not emerge in the separate treatment of the electric and magnetic contributions.

By using the vector identity

(3.11) C⋅(∇×A)−A⋅(∇×C)=∇⋅(A×C) ,

i.e.

(3.12) C⋅B−A⋅E=∇⋅(A×C) ,

we can rewrite the last integral of equation (3.9) as

(3.13) $$I_{int}=\int_{D}\nabla \cdot (A\times C)\;dV=\int_{S_{R}}(A\times C)\cdot dS\;,$$

where by the divergence theorem we can express the last integral as a surface integral defined over the spherical boundary $S_{R}=\partial D$ ($dS=\hat{\nu }\;dS$, with $\hat{\nu }$ outward unit normal to $S_{R}$). With reference to the Poynting vector momentum E×B, the interaction term given by

(3.14) $$I_{int}=-\int_{S_{R}}(C\times A)\cdot dS\;$$

represents the flux of the cross product C×A through $S_{R}$. For localized fields, this integral can be expressed in terms of the electric and magnetic cross-helicities used in the study of topologically non-trivial electromagnetic fields [28], in structured light, where the spin–orbit coupling of photons is important [22,25] and in more abstract, general settings [29].

### 3.2. Chirality under parity transformation

Under parity transformation, we have $H_{E}\mapsto -H_{E}$ and $H_{B}\mapsto -H_{B}$. The chiral character of the individual helicities holds true also for the RSH. Indeed, under parity transformation, we have $F\mapsto -F^{*}$ (and $F^{*}\mapsto -F$), so that

(3.15) $$H_{\text{RS}}=\frac{1}{2}(H_{F}+H_{F^{*}})\;\mapsto \;H_{\text{RS}}=\frac{1}{2}(-H_{F^{*}}-H_{F})=-H_{\text{RS}}\;.$$

As for the interaction term $I_{int}$, from the last equation (3.13), we have

(3.16) $$I_{int}=\int_{S_{R}}(A\times C)\cdot \hat{\nu }\;dS\;\mapsto \;\int_{S_{R}}-(A\times C)\cdot (-\hat{\nu })\;dS=I_{int,}$$

so that the interaction term remains unchanged under parity transformation.

### 3.3. Invariance under duality transformation

It is well known that Maxwell's equations in free space are invariant under duality transformation. This property holds true for the RSH as well.

**Lemma 1.**

*The RSH is invariant under duality transformation*.

**Proof.**

Consider the duality transformation under the rotation matrix $R_{\theta }$ defined by

(3.17) $$\left(\begin{array}{c} E^{\prime }\\ B^{\prime } \end{array}\right)=\left(\begin{array}{cc} \cos\theta & \sin\theta \\ -\sin\theta & \cos\theta \end{array}\right)\left(\begin{array}{c} E\\ B \end{array}\right)=R_{\theta }\left(\begin{array}{c} E\\ B \end{array}\right)\;.$$

From the definition of $E^{\prime }$ and $B^{\prime }$ in terms of the new potentials $C^{\prime }$ and $A^{\prime }$, we have

(3.18) $$\left\{ \begin{array}{clc} & C^{\prime }=A\sin\theta +C\cos\theta \;, & \;\\ & A^{\prime }=A\cos\theta -C\sin\theta \;. & \; \end{array}\right.$$

Direct computation of the transformed helicities gives

(3.19) $$\begin{array}{r} H_{E}^{\prime }=\int C^{\prime }\cdot E^{\prime }\;dV=\sin^{2}\theta \;H_{B}+\cos^{2}\theta \;H_{E}+\sin\theta \cos\theta \int (C\cdot B+A\cdot E)\;dV\; \end{array}$$

and

(3.20) $$\begin{array}{r} H_{B}^{\prime }=\int A^{\prime }\cdot B^{\prime }\;dV=\sin^{2}\theta \;H_{E}+\cos^{2}\theta \;H_{B}-\sin\theta \cos\theta \int (C\cdot B+A\cdot E)\;dV\;. \end{array}$$

The total helicity $H^{\prime }=H_{E}^{\prime }+H_{B}^{\prime }$ is given by

(3.21) $$\begin{array}{rl} H^{\prime }= & \;(\cos^{2}\theta +\sin^{2}\theta )\;H_{B}+(\cos^{2}\theta +\sin^{2}\theta )\;H_{E}\\ & +\sin\theta \cos\theta \int (C\cdot B+A\cdot E)\;dV-\sin\theta \cos\theta \int (C\cdot B+A\cdot E)\;dV\\ = & \;H_{B}+H_{E}\;. \end{array}$$

Hence, from the definition (equation (3.7)), we have that under duality transformation, the RSH remains unchanged.

A fundamental aspect of $H_{F}$ is its direct connection with the symmetry of Maxwell's equations in free space. In the absence of sources, Maxwell's equations reduce to

(3.22) ∇⋅E=0 ,∇⋅B=0 

and

(3.23) $$\nabla \times E=-\partial_{t}B\;,\;\nabla \times B=\partial_{t}E\;,$$

which are invariant under electromagnetic duality transformations, mixing the electric and magnetic fields as

(3.24) E↦Ecos⁡θ+Bsin⁡θandB↦−Esin⁡θ+Bcos⁡θ .

In the RS context, this symmetry corresponds to a phase rotation of the RS vector, given by

(3.25) $$F\;\mapsto \;e^{-i\theta }F\;.$$

Taking the same transformation for the complex potential $G\;\mapsto \;e^{-i\theta }G$, we have

(3.26) $$H_{F}=\int G^{*}\cdot F\;dV\;,$$

which remains unchanged under such transformation. This simply demonstrates that the properties of $H_{F}$ are directly inherited from the symmetry of Maxwell's equations in free space, showing that $H_{F}$ is a natural quantity in the dual-symmetric formalism.

Note that the electric and magnetic helicities are not separately invariant under duality transformations, but mix in such a way that their sum $H_{RS}=H_{E}+H_{B}$ remains unchanged. This indicates that the decomposition into separate contributions is not inherently compatible with the fundamental symmetry of the theory, reinforcing the interpretation of $H_{F}$ as a single, individual entity. Here, $H_{F}$ can thus be seen as a generalization of classical helicity that not only unifies the electric and magnetic helicity contents, but it also adapts to the symmetry structure of Maxwell's equations in free space. By direct computation, one can then verify that $H_{F^{*}}$ is also conserved under duality, and the same holds true for the interaction term:

(3.27) $$\begin{array}{rl} I_{int}^{\prime }= & \int_{S_{R}}(A^{\prime }\times C^{\prime })\cdot dS=\int_{S_{R}}[(A\cos\theta -C\sin\theta )\times (A\sin\theta +C\cos\theta )]\cdot dS\\ = & \int_{S_{R}}(A\times C)\cdot dS=I_{int}\;. \end{array}$$

The imaginary part of $H_{F}$, given by

(3.28) Im(HF)=∫(C⋅B−A⋅E)dV 

presents peculiar properties with respect to the fundamental symmetries of electromagnetic theory, because under parity transformations Im(HF) is even. This indicates that the coupling term is not related to the chirality of the field. On the other hand, under electromagnetic duality transformation, the complex functional $H_{F}$ remains unchanged. This behaviour highlights that the decomposition into real and imaginary parts is not intrinsically privileged from the point of view of fundamental symmetries, and that the coupling term plays an essential role in the dual-symmetric structure of the functional. It therefore emerges as a non-trivial consequence of the adopted complex formalism and has no direct analogue in the classical, separate treatment of the electric and magnetic components.

## 4. Electric field as a material field

Let us now consider the case of null fields in ideal conditions, in the absence of free charges (∇⋅E=0) and currents (J=0). Null fields are prescribed by taking

(4.1) E⋅B=0andE⋅E=B⋅B≠0 

with Lorentz invariants $I_{1}=E\cdot B$ and $I_{2}=\|E{\|}^{2}-\|B{\|}^{2}$ (see also §6) vanishing simultaneously. Under these conditions, the electric field behaves as a material, frozen-in field. To show this, let us recall the following result [30,31]:

**Lemma 2 (Helmholtz–Żorawski criterion).**

*Let* a=a(x,t) *be a vector field transported by a flow field* u=u(x,t) *in an unbounded domain* $D\subseteq R^{3}$; a*is a material field if it satisfies the equation*

(4.2) $$[\frac{\partial a}{\partial t}+\nabla \times (a\times u)+u(\nabla \cdot a)]\times a=0\;.$$

Let us take a≡E: in order to ensure that E is a material field, we must have the term in the square bracket above […]=fE (where f=f(x) is a scalar function of the position). Here, we simply assume f=0, restricting ourselves to the stronger condition of electric flux conservation, which implies field line conservation. This is consistent with the assumption that in the absence of sources and currents, the electric field is simply subject to the kinematic transport by u.

In the absence of free charges, the last term in the square brackets is identically zero, and in the absence of currents, Ampère's equation reduces to

(4.3) $$\frac{\partial E}{\partial t}=\nabla \times B\;.$$

Using equation (4.3), equation (4.2) simplifies to

(4.4) ∇×(u×E)−∇×B=0 .

Now, in order to ensure that E is a material field, we consider the Poynting flow that guarantees the frozenness condition for E. For this, let us take u=E×B/(B⋅B), and substitute the normalized Poynting vector into equation (4.4); we have

(4.5) $$\nabla \times (\frac{E\times B}{B\cdot B}\times E)-\nabla \times B=0\;.$$

Using the standard vector identity for the triple product, equation (4.5) becomes

(4.6) $$\nabla \times (\frac{B(E\cdot E)-E(E\cdot B)}{B\cdot B})-\nabla \times B=0\;.$$

Thus, under the null fields condition, equation (4.1), we have

(4.7) $$\nabla \times B-\nabla \times (\frac{E(E\cdot B)}{B\cdot B})-\nabla \times B=0\;,$$

which is identically verified; hence, the electric field under the null conditions (equation (4.1)) is a material field in free space.

## 5. Conservation of the Riemann–Silberstein helicity

In the absence of free charges and currents, null fields propagate in the vacuum (locally) as radiation fields. We have the following.

**Theorem 1 (Electric helicity conservation).**

*Let* E *and* B *be localized null fields in an unbounded domain* $D\subseteq R^{3}$. *In the absence of free charges* (∇⋅E=0) *and currents* (J=0), *we have*

(5.1) $$\frac{dH_{E}}{dt}=\frac{d}{dt}\int C\cdot E\;dV=0\;\Rightarrow \;H_{E}=constant.$$

**Proof.**

Let us rewrite Ampère's equation in terms of the electric vector potential C; we have

(5.2) $$\frac{\partial }{\partial t}(\nabla \times C)=\nabla \times \frac{\partial C}{\partial t}=\nabla \times B\;.$$

By uncurling the left-hand side of equation (5.2), we get

(5.3) $$\frac{\partial C}{\partial t}=B+\nabla \psi \;.$$

From the definition of electric helicity, and relying on the material property of the electric and magnetic field, we have

(5.4) $$\frac{dH_{E}}{dt}=\int (\frac{\partial C}{\partial t}\cdot E+C\cdot \frac{\partial E}{\partial t})\;dV=\int [(B+\nabla \psi )\cdot E+C\cdot \frac{\partial E}{\partial t}]\;dV\;,$$

where in the last integral we have used equation (5.3). Again, from Ampère's law

(5.5) $$C\cdot \frac{\partial E}{\partial t}=C\cdot (\nabla \times B)\;,$$

and upon using the vector identity

C⋅(∇×B)=∇⋅(B×C)+B⋅E ,

we can rewrite the left-hand side of equation (5.4) as

(5.6) $$\frac{dH_{E}}{dt}=\int [2(B\cdot E)+(\nabla \psi )\cdot E+\nabla \cdot (B\times C)]\;dV=\int [(\nabla \psi )\cdot E+\nabla \cdot (B\times C)]\;dV\;,$$

because by assumption B⋅E=0. Integrating by parts the first term on the right-hand side of equation (5.6), we get

(5.7) ∫(∇ψ)⋅EdV=∫∇⋅(ψE)dV−∫ψ(∇⋅E)dV .

Since in the absence of charges ∇⋅E=0, the last term above vanishes. Applying the divergence theorem to the first term on the right-hand side above, we have

(5.8) $$\int_{D}(\nabla \psi )\cdot E\;dV=\int_{S_{R}}\psi \;E\cdot dS\;.$$

Under the assumed decay of E at infinity, this surface term vanishes; hence, the gauge contribution from ∇ψ has no effect on $dH_{E}/dt$. Moreover, by taking the Coulomb gauge ∇⋅C=0, we obtain $\nabla^{2}\psi =0$ (harmonic freedom) which, under the usual boundary conditions, also vanishes.

As for the integral of the triple product in equation (5.6), we have

(5.9) $$\frac{dH_{E}}{dt}=\int_{D}\nabla \cdot (B\times C)\;dV=\lim_{R\to \infty }\int_{S_{R}}(B\times C)\cdot dS=0\;,$$

because B is localized, decaying sufficiently fast at infinity to make the integral convergent.

For completeness, let us include also a brief proof of the conservation of magnetic helicity in free space.

**Lemma 3 (Magnetic helicity conservation).**

*Let* E *and* B *be localized null fields in an unbounded domain* $D\subseteq R^{3}$. *In the absence of free charges* (∇⋅E=0) *and currents* (J=0), *we have*

(5.10) $$\frac{dH_{B}}{dt}=\frac{d}{dt}\int A\cdot B\;dV=0\;\Rightarrow \;H_{B}=constant.$$

**Proof.**

From the definition of magnetic helicity, and relying on the material property of the electric and magnetic field, we have

(5.11) $$\frac{dH_{B}}{dt}=\int (\frac{\partial A}{\partial t}\cdot B+A\cdot \frac{\partial B}{\partial t})\;dV\;.$$

Using the Faraday gauge relation and the condition E⋅B=0, we have

(5.12) $$\frac{\partial A}{\partial t}\cdot B=-E\cdot B-B\cdot \nabla \phi =-B\cdot \nabla \phi \;.$$

Applying Faraday's law ∂B/∂t=−∇×E, we get

(5.13) $$A\cdot \frac{\partial B}{\partial t}=-A\cdot (\nabla \times E)\;,$$

and using the vector identity A⋅(∇×E)=E⋅(∇×A)−∇⋅(A×E), we have

(5.14) $$A\cdot \frac{\partial B}{\partial t}=-E\cdot B+\nabla \cdot (A\times E)=\nabla \cdot (A\times E)\;.$$

Since ∇⋅B=0, substituting the right-hand sides of equations (5.12) and (5.14) into equation (5.11), we have

(5.15) $$\frac{dH_{B}}{dt}=\int_{D}\nabla \cdot (-\phi B+A\times E)\;dV=\int_{S_{R}}(-\phi B+A\times E)\cdot dS=0\;,$$

because of the assumed fast decay of the fields.

An immediate consequence of the result above is the following.

**Corollary 1 (RSH conservation).**

*The RSH* $H_{\text{RS}}$ *of the null fields* E *and* B *in free space is conserved in* D.

Note that in the absence of sources (and under the assumed fast decay of the fields at infinity), the null condition E⋅B=0 is sufficient to guarantee the conservation of both electric and magnetic helicities. Note also that the invariance of RSH under duality transformation (lemma 1) is consistent with its interpretation as a Noether charge [32].

## 6. A note on the Faraday tensor formulation

The results above admit a natural covariant formulation in terms of the Faraday tensor $F^{\mu \nu }$ defined in the metric signature (+,−,−,−) by

(6.1) $$F^{\mu \nu }=\left(\begin{array}{cccc} 0 & -E_{x} & -E_{y} & -E_{z}\\ E_{x} & 0 & -B_{z} & B_{y}\\ E_{y} & B_{z} & 0 & -B_{x}\\ E_{z} & -B_{y} & B_{x} & 0 \end{array}\right)$$

and its Hodge dual ${}^{⋆\;}F^{\mu \nu }=(\epsilon^{\mu \nu \rho \sigma }F_{\rho \sigma })/2$. As mentioned in §4, for null fields the two Lorentz invariants $I_{1}=F_{\mu \nu }{}^{⋆\;}F^{\mu \nu }$ and $I_{2}=F_{\mu \nu }F^{\mu \nu }$ vanish identically. Note that the distinguished transport velocity given by the Poynting flow is not externally prescribed, but is intrinsically encoded in the covariant formalism through the electromagnetic stress-energy tensor

(6.2) $$T^{\mu \nu }=-F^{\mu \alpha }F_{\alpha }^{\nu }+\frac{1}{4}\eta^{\mu \nu }F_{\alpha \beta }F^{\alpha \beta }\;,$$

where $T^{0i}=S^{i}$ is just the component of S=E×B.

A covariant interpretation of helicity conservation may be obtained by introducing the magnetic and electric helicity currents

(6.3) $$J_{E}^{\mu }=\frac{1}{2}\epsilon^{\mu \nu \rho \sigma }C_{\nu }{}^{⋆\;}F_{\rho \sigma }\;\text{and}\;J_{B}^{\mu }=\frac{1}{2}\epsilon^{\mu \nu \rho \sigma }A_{\nu }F_{\rho \sigma }\;,$$

whose temporal components reduce, respectively, to the standard electric and magnetic helicity densities

(6.4) $$J_{E}^{0}=C\cdot E\;\text{and}\;J_{B}^{0}=A\cdot B\;.$$

A direct computation gives

(6.5) $$\partial_{\mu }J_{E}^{\mu }=-\frac{1}{2}F_{\mu \nu }{}^{⋆\;}F^{\mu \nu }\;\text{and}\;\partial_{\mu }J_{B}^{\mu }=\frac{1}{2}F_{\mu \nu }{}^{⋆\;}F^{\mu \nu }\;.$$

Therefore, for null electromagnetic fields satisfying $F_{\mu \nu }{}^{⋆\;}F^{\mu \nu }=0$, both helicity currents are divergence-free, i.e.

(6.6) $$\partial_{\mu }J_{E}^{\mu }=\partial_{\mu }J_{B}^{\mu }=0\;.$$

Under the assumed fast decaying fields, we have then

(6.7) $$\frac{dH_{E}}{dt}=\frac{dH_{B}}{dt}=0\;,$$

showing that the electric and magnetic helicities are separately conserved. The helicity-preserving properties discussed above are thus a direct consequence of the vanishing pseudoscalar invariant $F_{\mu \nu }{}^{⋆\;}F^{\mu \nu }$.

## 7. Concluding remarks

The time invariance of the RSH can be traced back to the work of Bliokh *et al.* [22], where RSH is considered in the context of the dual-symmetric formulation of electromagnetism, and the work of Elbistan *et al.* [32], where RSH is obtained as a Noether charge from a Lagrangian formulation. Here, we have provided a direct proof of its time invariance by demonstrating the conservation of the electric and magnetic helicities for radiation fields in free space. We have also demonstrated that the RSH is chiral under parity transformation and remains invariant under duality, concluding with a brief re-elaboration of these results in terms of Faraday's tensor and covariant formulation.

With the present work, we provide a coherent and unified presentation for the conservation of electromagnetic helicity that may find applications for future studies of topologically complex field structures [15,16,33], including current works on Hopfion solutions in electromagnetic fields [17–21], optical properties in structured light [34], and the interactions of radiation fields and matter for advanced technological applications [35].

## Data Availability

This article has no additional data.
